# The Effect of Submucosal Injection of Plasma-Rich Platelets on Blood Inflammatory Markers for Patients with Bimaxillary Protrusion Undergoing Orthodontic Treatment

**DOI:** 10.1155/2019/6715871

**Published:** 2019-10-01

**Authors:** Trefa Mohammed Ali Mahmood, Omar Fawzi Chawshli

**Affiliations:** ^1^Department of Pedodontics, Orthodontics and Preventive Dentistry, College of Dentistry, University of Sulaimani, Sulaymaniyah, Kurdistan Region, Iraq; ^2^Department of Pedodontics, Orthodontics and Preventive Dentistry, College of Dentistry, Hawler Medical University, Erbil, Kurdistan Region, Iraq

## Abstract

**Objectives:**

The present study aims to reveal the systemic effects of submucosal injection of plasma-rich platelets (PRP) on blood inflammatory markers which was used in an attempt to reduce the retraction time of the upper canine following extraction of upper maxillary premolars for patients with bimaxillary protrusion.

**Hypothesis:**

No change on comparing the values of blood inflammatory markers before and after submucosal injection of PRP.

**Methods:**

Eighteen female patients with bimaxillary protusion were selected from patients seeking orthodontic treatment from the College of Dentistry/University of Sulaimai, whose maxillary and mandibular first premolars were decided to be extracted after proper diagnosis. Thirty-three blood markers (twenty hematological and thirteen biochemical markers) were estimated before orthodontic bracketing, 24 hours and 7 days following submucosal injection of PRP (5 cc) to reveal the systematic effect of PRP on blood inflammatory markers that were used in an attempt to reduce the retraction time of the upper canine following extraction of upper maxillary premolars for patients with bimaxillary protrusion.

**Results:**

The results indicate nonsignificant differences in the values of all blood markers except for gamma GT (GGT), PDWa, serum albumin, serum total protein, and total calcium. Gamma level significantly increased for both test intervals. On the other hand, there was a significant drop in the value of PDWa while for alkaline phosphatase, there was a drop within the first 24 hr of PRP injection while after 7 days the value was significantly increased. On the other hand, there was a drop in the level of serum albumin, while there was an increase in the serum total protein and total calcium.

**Conclusion:**

Submucosal injection of PRP could lead to systematic alteration of blood parameters including ALK phosphatase, gamma GT, serum albumin, and serum total protein, which may be related to liver function in addition to increase in the level of PDWa and serum calcium. We present evidence that PRP contains and may trigger systemic effect. Thus, further investigation is recommended to follow up the patient for a longer period of time and on a larger sample. This trial is registered with U1111-1221-8829 by Sri Lanka Clinical Trial Registry, SLCTR/2018/040, and No. 64 on 6^th^ August 2018 at the local clinical studies database, College of Dentistry.

## 1. Introduction

Since the inception of the practice of orthodontics, one of the domains in the research has been the tooth movement and associated biological reactions. Research has been done to study various approaches to achieve tooth movement with most physiological manner but with maximum pace [1].

PRP has recently been considered as an orthobiological adjuvant treatment [2], currently used in different medical fields. The interest in the application of PRP in dermatology has recently increased as it is being used in several different applications such as in tissue regeneration, wound healing, scar revision, skin rejuvenating effects, and alopecia [3]. PRP has the potential and capability to promote periodontal regeneration through various mechanisms. The effect of PRP in localized acceleration of tooth movement is dependent on the concentration used. However, the method of synthesis is critical to the success of PRP-based acceleration of tooth movement. The use of injectable PRP at a different stage of orthodontic treatment can improve the quality of the treatment outcome by influencing the bone quality and enhancing the rate of tooth movement [1].

PRP is defined as an autologous concentration of platelets in a small volume of plasma and is considered to be a rich source of autologous growth factors (GFs) [4]. GFs are natural biologic mediators that regulate key cellular events that are part of the process of tissue repair and regeneration. After binding of GFs to specific cell membrane receptors of target cells, intracellular signaling pathways are induced; this typically results in the activation of genes that may ultimately change the cellular activity and phenotype. However, the effect of each GF is regulated through a complex system of feedback loops, which involve other GFs, enzymes, and binding proteins. Recent advances in the areas of cellular and molecular biology have allowed better understanding of the functions of GFs. *In vitro* and *in vivo* studies have confirmed that GFs can enhance the capacity of tissues to regenerate by regulating cell chemoattraction, differentiation, and proliferation [5].

PRP components interact with cells involved in the immune response and inflammation, angiogenesis, cell migration and differentiation, and anabolism and catabolism of the extracellular matrix. This list of PRP elements is not comprehensive: interleukin 1b (1b); tumor necrosis factor (TNF); platelet-derived growth factor (PDGF); tissue growth factor (TGF); vascular endothelial growth factor (VEGF); and fibroblastic growth factor (FGF) [6]. There are several systemic biomarkers that could be related to orthodontic treatment. For instance, according to Yashin et al. [7], there were significant increases in hs-CRP level, WBC count, and neutrophil count while a significant decrease in Na level. K level was significantly decreased on day one. Indicating a systemic immune response develops against therapy in patients undergoing fixed orthodontic therapy. Ileri et al. [8] concluded that piezocision procedure might be related to transitory bacteremia. Hence, orthodontists should consider the possibility of bacterial endocarditis in at-risk patients when piezocision is part of the treatment plan, while Azeem et al. [9] revealed that the micro-osteoperforation technique is not related to transitory bacteremia.

## 2. Methodology

This study was conducted in the Department of Pedodontics, Orthodontics, and Preventive Dentistry, College of Dentistry, University of Sulaimani, with the corporation Shar Medical Center Library.

### 2.1. The Sample

Eighteen females with bimaxillary protrusion were selected from patients seeking orthodontic treatment, whose maxillary and mandibular first premolars were decided to be extracted after proper diagnosis using study models, digital cephalomerty, orthopantomograph, and CBCT; all the cases were evaluated with the supervisor (Orthodontist).

The Simplified Oral Hygiene Index (OHI-S) was used to estimate the oral health of the selected patients which is different from the original Oral Hygiene Index (OHI) in the number of the tooth surfaces scored (6 rather than 12), the method of selecting the surfaces to be scored, and the scores which can be obtained. The criteria used for assigning scores to the tooth surfaces are the same as those used for the OHI.

The OHI-S, like the OHI, has two components: the Debris Index and the Calculus Index. Each of these indexes, in turn, is based on numerical determinations representing the amount of debris or calculus found on the preselected tooth surfaces [10]. Zero oral hygiene indices were scored for all participants prior to begin the sequences of treatment.

### 2.2. Design

Experimental study (split mouth) was employed in this study. Patients were considered eligible for the study if they meet the following inclusion criteria:

1. Aged between 18 and 26 years
2. Bimaxillary protrusion
3. Minimum crowding (less than 2 mm) or minimum spacing (less than 4 mm)
4. Indication for extraction of upper and lower first premolars
5. The feasibility of bonding brackets
6. No previous orthodontic treatment
7. No systemic diseases
8. No smoking
9. Good oral hygiene

The exclusion criteria were as follows:

1. Patients with severe tooth displacement (e.g., ectopic canine)
2. Those reporting the use of medications throughout the study

The rights of patients were protected and the purpose and methods of the study were completely explained to the patients and parents; informed consent was obtained from each blood inflammatory marker. Blood samples (5 ml) were drawn at the baseline on day zero, and after 24 hours of acceleration (beginning retraction) [11], the following blood tests were performed [7]:

1. C-reactive protein (CRP)
2. CBC parameters
3. Levels of aspartate aminotransferase (AST)
4. Alanine aminotransferase (ALT)
5. Gamma glutamyl transferase (GGT)
6. Alkaline phosphatase (ALP)
7. Urea
8. Creatinine
9. Sodium (Na)
10. Potassium (K)
11. Calcium (Ca)
12. Total protein (TP)
13. Albumin (Alb)

These tests were performed at Shar Medical Center laboratory before acceleration (Tb0) and 24 hours (Tb1) and 7 days (Tb7) following acceleration.

## 3. Clinical Procedure

Five to seven days after first premolars extraction, fixed orthodontic appliances of MBT prescription 0.022-inch slot height were bonded. Then, a 0.014-inch NiTi archwire was inserted and tied to each bracket using ligature wires.

Arch wire sequences used were 0.014-inch NiTi followed by 0.018 inch NiTi, 0.017–0.025 inch NiTi, and finally 0.017–0.025 stainless steel. Before retraction self-drilling temporary anchorage devices of 10 mm length and 1.6 mm diameter were inserted with hand drill between the upper second premolar and the upper first molar for both sides as an anchorage for retraction force, as well as for the lower arch.

At this stage, upper canines were retracted with the use of maximum anchorage (TADs). The right side composed the study group, whereas the left side served as the control group.

The retraction phase was initiated after PRP injection on the experimental side (right), using elastomeric chains with a force of 150 gm, translation movement according to Kanuru et al. [12], measured using stress and tension gauge dial type (Dentaurum).

For the control side, retraction was started at the same time with the same mechanics. Patients were examined at two week intervals, and the elastomeric chains were replaced at each appointment until ideal class I canine relationships were established (bracket system).

**Figure 1:**
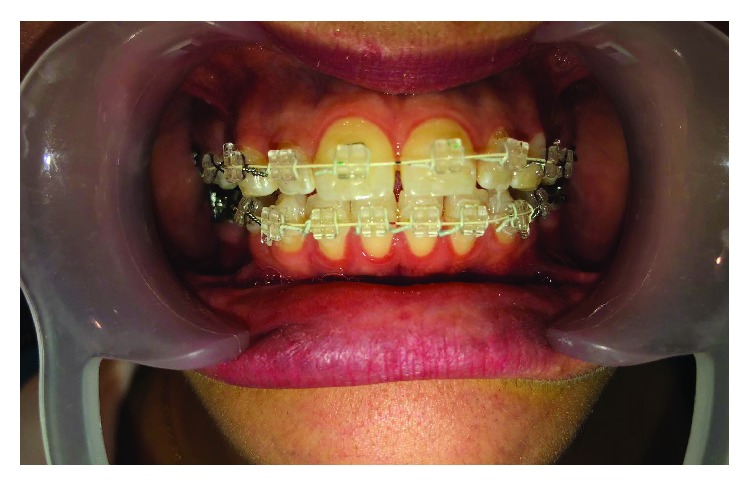
Bracketing.

Ultraesthetic brackets, archwires, and accessories were used; sapphire bracket (MBT Slot 0.022 inch slot height) from DW Orthoworld Company, which totally blends with the dental structure, was used. Mimetic is designed with advanced 3D technology to offer great adaptation and comfort for the patient. Bonding was with OrthoFlow compsite of the same company that does not require any bonding agent on the enamel surface in order to meet both clinical and aesthetic needs (Figure 1).

## 4. PRP Preparation

**Figure 2:**
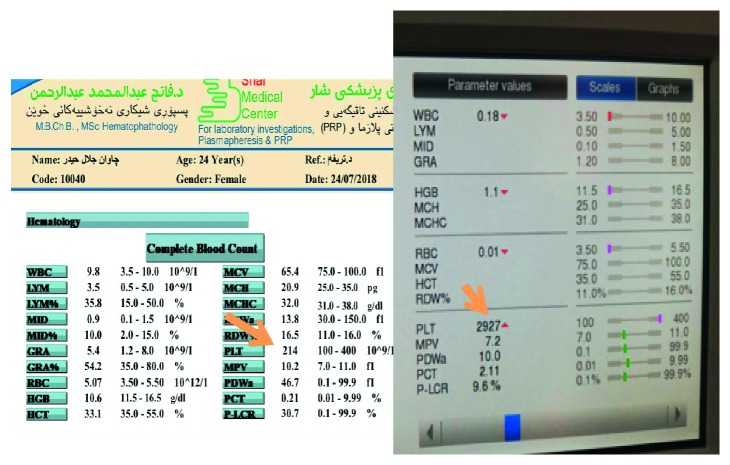
ROTIXA 500 RS Hettich floor-standing centrifuge was used, producing high concentration PRP (5 times the concentration in whole blood).

The variation of platelets and other blood component concentrations between commercial PRP kits may affect clinical treatment outcomes [13]. A 30 cc venous blood draw will yield 3–5 cc of PRP [14]. There are many ways of preparing PRP. It can be prepared by the PRP method or by the buffy-coat method. In this study, we will use the PRP method, using an initial centrifuge to separate red blood cells (RBC) followed by a second centrifuge to concentrate platelets, which are suspended in the smallest final plasma volume. Blood is initially collected in PRP tubes that contain anticoagulant citrate dextrose (ACD). The first spin step is performed at constant acceleration to separate RBCs from the remaining blood volume for 9 minutes at about 2000 rpm. After the first spin step, blood will separate into three layers: an upper layer that contains mostly platelets and WBC, an intermediate thin layer that is known as the buffy coat and rich in WBCs, and a bottom layer that consists mostly of RBCs. For the production of pure PRP, the upper layer and superficial buffy coat are transferred to an empty sterile tube, and the second spin step is then performed for 10 minutes at 3870 rpm; thus, the lower 1/3rd will be the PRP (platelet-rich plasma). So, the procedure, in detail, involves a 35 cc of venous blood draw using aseptic technique from median cubital vein of the patient. A butterfly needle was used in efforts of avoiding irritation and trauma to the platelets, which are in a resting state. ROTIXA 500 RS Hettich floor-standing centrifuge (Figure 2), producing high concentration PRP (5 times the concentration in whole blood), an initial centrifuge to separate red blood cells (RBC) is followed by a second centrifuge to concentrate platelets, which are suspended in the smallest final plasma volume. Blood is initially collected in PRP tubes that contain anticoagulant citrate dextrose (ACD). The first spin step is performed at constant acceleration to separate RBCs from the remaining blood volume for 9 minutes at about 2000 rpm. After the first spin step, blood will separate into three layers: an upper layer that contains mostly platelets and WBC, an intermediate thin layer that is known as the buffy coat and rich in WBCs, and a bottom layer that consists mostly of RBCs. For the production of pure PRP, upper layer and superficial buffy coat are transferred to an empty sterile tube, and the second spin step is then performed for 10 minutes at 3870 rpm; thus, the lower 1/3rd will be the PRP (platelet-rich plasma).

## 5. Site of Injection

**Figure 3:**
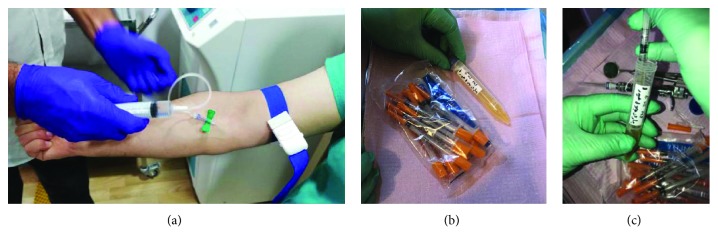
PRP preparation.

**Figure 4:**
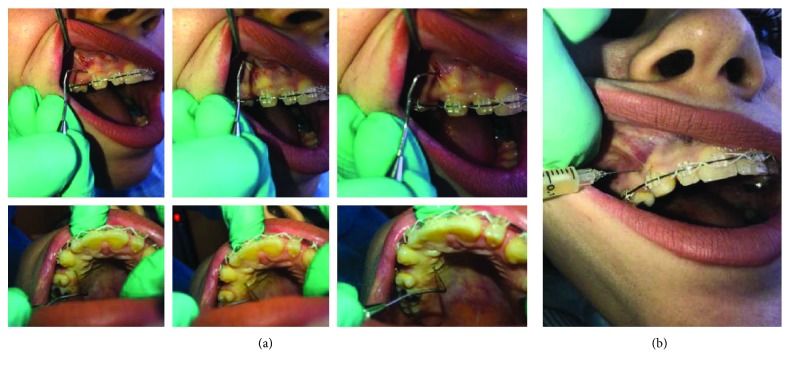
Site of PRP injection and force application.

Five cubic centimeters of PRP was injected by means of a microsyringe into the buccal and palatal vestibular mucosa distally through the attached gingiva into the oral mucosa to the root of the upper right canines of each patient under local anesthesia. All injections were volumetrically equivalent. Injections were performed only once on day zero of retraction and not repeated again (as illustrated in Figures 3 and 4).

Before the injection of PRP, local anesthesia (Xylocaine) was used at the target sites for *t* pain control. It is a submucosal injection rather than a subperiosteal injection. It is just similar to the injection of local anesthesia, and it has no certain injection pattern (six injections, each one was 0.8 cc). Acetaminophen (500 mg) was prescribed for the postinjection pain control. Nonsteroidal antiinflammatory drugs will neutralize the effects of PRP and were not used for the postinjection pain control [15].

## 6. Measurements

1. Blood parameter test before acceleration (Tb0), following 24 hours of acceleration (Tb1) and 7 days following acceleration (Tb7). Values were analyzed using the SPSS (Statistical Package for Social Science) for Windows.
2. Descriptive statistics consisting of mean, standard deviation (SD), and minimum (Min) and maximum (Max) for all the values at Tb0, Tb1, and Tb7 (Table 1).
3. One-way repeated measures analysis of variance was used, and *F*-ratio was used to compare the 3 groups of variables (Table 2).

## 7. Results

Descriptive statistical analysis including mean, minimum, maximum, and standard deviation is summarized in Table 1. The results indicate nonsignificant differences in the values of all blood markers except for PDWa, gamma GT (GGT), ALK phosphatase, S. albumin, S. total protein, and total calcium.

The gamma level significantly increased from 13.1 IU/L to 22.1 IU/L at Tb1 and 23.8 IU/L at Tb7 (*p* value 0.00001). On the other hand, there was a significant drop in the value of PDWa from 21.3 fl to 12.3 fl at Tb1 and 12.4 fl at Tb7 (*p* value 0.018), while for alkaline phosphatase there was a drop within the first 24 hr after PRP injection from 56.1 IU/L to 49.1 IU/L at Tb1, while after 7 days the value was significantly increased to 58.7 IU/L (*p* value 0.00001).

On the other hand, there was a drop in the level of serum albumin from 4.48 g/dl to 3.98 g/dl at Tb1 and 4.01 g/dl at Tb7 (*p* value 0.0035), while there was an increase in the serum total protein from 7.08 g/dl to 9.44 g/dl at Tb1 and 9.02 g/dl at Tb7 (*p* value 0.00001). Again, for the total calcium level, there was a significant increase from 9.3 mg/dl to 9.59 mg/dl at Tb1 and 9.65 mg/dl at Tb7 (*p* value 0.049), although all the values remain within the normal level except for serum albumin as shown in Table 2.

## 8. Discussion

Only females were included in this study, as 65% of patients seeking orthodontic treatment were females according to a study done in Sulaimani City by Amin et al., which also comes in accordance with other studies [16], also in an attempt to reduce bias related to biological responses that differ between genders.

As previous studies revealed that conventional orthodontic treatment is not associated with systemic immune response at any time points; for this reason, we do not include a control group in an attempt to reduce time and effort [17]. Besides, another study assessed the effects of fixed orthodontic therapy on high-sensitivity C-reactive protein (hs-CRP) level, CBC parameters, and levels of aspartate aminotransferase (AST) and alanine aminotransferase (ALT), gamma glutamyl transferase (GGT), alkaline phosphatase (ALP), urea, creatinine, sodium (Na), potassium (K), calcium (Ca), total protein (TP), and albumin (Alb), and their results confirm that an elevation in serum hs-CRP levels and neutrophil-lymphocyte ratio within first 3 months [18] does not have effects on the blood parameters that were assessed in our study (PDWa, gamma GT (GGT), ALK phosphatase, S. albumin, S. total protein, and total calcium). Thus, it gives a proof that our result is solely related to PRP injection.

PRP is injected submucosally not subperiosteally following the standardization and the proposal of the use of PRP in orthodontics according to previous studies [15].

Starting with gamma-glutamyl transferase (GGT), there was a significant increase for both Tb1 and Tb7 within the normal range, but still it is an interesting finding especially when it comes with significant decrease of serum albumin level.However, GGT's predictive utility applies well beyond liver disease, and elevated GGT is linked to increased risk to a multitude of diseases and conditions, including cardiovascular disease, diabetes, metabolic syndrome, and all-cause mortality [19].

Low antioxidant defenses are also correlated with elevated GGT [20]. GGT is an enzyme found in cell membranes of many tissues mainly in the liver, kidney, and pancreas. It is also found in other tissues including the intestine, spleen, heart, brain, and seminal vesicles. The highest concentration is in the kidney, but the liver is considered the source of normal enzyme activity [21].

Secondly, the red cell distribution width (RCDW) test is used to study the distribution of RBCs not their actual size. Levels outside of the normal range can indicate conditions such as anemia, malnutrition, and liver disease [21].

Thirdly, the alkaline phosphatase (ALP) level in healthy adults should be 20–140 U/L. As ALP is most abundant in the bones and liver, elevated ALP levels are generally a sign of a liver or bone condition. An obstruction of the liver or damage to it will cause ALP levels to rise. This will also occur if there is an increase in bone cell activity [22].

Fourthly, the normal range of serum albumin is 3.5 to 5.2 g/dl. Serum albumin measures the amount of albumin in the clear liquid portion of blood. Conditions associated with “low” levels of albumin are as follows: ascites, burns, glomerulonephritis, liver disease (hepatitis or cirrhosis), malabsorption syndrome (e.g., Crohn's disease, celiac disease, or Whipple disease), and malnutrition. Serum albumin is a multifunctional circulatory protein, and its concentration is influenced by several factors including its synthesis rate, catabolism rate, extravascular distribution, and exogenous loss. Moreover, both nutritional status and systemic inflammation affect the synthesis of serum albumin. It is of interest to understand the prognostic value in the full spectrum of cardiovascular disease in the era of newly developed pharmacological and interventional treatments. As illustrated in the results, it is significantly dropped after 24 hours following submucosal injection of PRP [23], as albumin production may be inhibited by proinflammatory mediators [24].

Fifthly, serum proteins are mainly synthesized in the liver and, among other functions, maintain blood volume through the colloidal osmotic effect, buffer blood pH, transport hormones and drugs, participate in cell coagulation, catalyze chemical reactions (enzymes), regulate the metabolism (hormones), and participate in the body's defense against foreign agents [25]. A rise in protein levels is noted in dehydration, amyloidosis, and chronic inflammatory states [26].

Alongside the total serum protein level, the albumin to globulin (A/G) ratio in the bloodstream can be calculated in a laboratory. This is because some conditions affect the amounts of albumin or globulin in the blood. A low A/G ratio may be due to an overproduction of globulin, underproduction of albumin, or loss of albumin, which may indicate the following: an autoimmune disease, cirrhosis involving inflammation and scarring of the liver, multiple myeloma, and nephrotic syndrome, a kidney disease [27].

Lastly, calcium concentration is characterized by a high physiological variation, depending on age, sex, physiological state, and even season (owing to the seasonal variation of vitamin D, which is directly involved in the regulation of calcium concentration). Unless serum proteins contain abnormalities, total serum calcium concentration is normally between 8.5 and 10.2 mg/dl of serum. Because ionized calcium is the only component of the total serum calcium level that is regulated by calciotropic hormones, decisions on the total serum calcium concentration should not be made unless changes in concentrations of plasma proteins, particularly albumin, are considered [28]. So, a common cause of elevated serum calcium is secondary to increased serum binding protein. Calcium levels are dictated by the actions of parathyroid hormone (PTH), calcitonin, and calcitriol. PTH levels rise and fall in response to serum calcium levels. High levels of PTH stimulate a rise in serum calcium by increasing both renal tubular calcium reabsorption and bone resorption. PTH also stimulates the conversion of calcidiol to calcitriol in the kidneys. Calcitriol leads to a further increase in serum calcium via increased absorption of calcium in the small intestine. Phosphate metabolism is also controlled by PTH and calcitriol; PTH generally lowers phosphate levels through its effects on the kidney, while calcitriol generally raises phosphate levels through its effects on the intestine and inhibitory effects on PTH levels. Liver recovery is an extremely complicated process that involves intercellular interaction between growth factors and cytokines [28].

No previous study was associated with the effect of PRP on the liver. Salem et al. described the regenerative impact of platelets in the liver of adult male Wistar rats, which comprises three pathways: a direct impact on hepatocytes, a favorable impact on liver sinusoidal endothelial cells, and a collaborative impact on Kupffer cells [29]. Therefore, it was proposed that the expansion of platelets induced by platelet transfusion would enhance liver functions in patients with chronic liver diseases in the clinical setting [30]. Growth factors and cytokines cause activation of downstream cascades, related to the advancement of quiescent hepatocytes into the cell cycle.

All the blood parameters that have been significantly altered after submucosal injection of PRP are related to liver function; hereby, we can correlate these findings to the following: although PRP is locally injected, it has systematic influence by induction of inflammation of vital structures such as the liver and kidney which gives rise to a serious question: can PRP cause systematic adverse effects? However, PRP is locally used in dentistry or for dermatological purposes. Again, a question concerning patients already having liver problems arises: are they suitable for PRP therapy? So, further investigation is needed for a larger sample size within different age groups to verify the systematic effect of local PRP injection for a longer duration and repeated injection cases.

## 9. Conclusion

Submucosal injection of PRP could lead to systematic alteration of blood parameters including ALK phosphatase, gamma GT, S. albumin, and S. total protein, which may be related to liver function impairment, in addition to increase in the level of PDWa and calcium.

## Figures and Tables

**Table 1:** Descriptive statistical analysis.

Blood markers	Initial (Tb0)				After 24 hr (Tb1)				After 7 days (Tb7)			
	Mean	Min	Max	SD	Mean	Min	Max	SD	Mean	Min	Max	SD
WBC	7.57	3.4	11.5	2.4	7.7	5.2	10.7	1.79	7.63	5.1	10.1	1.47
LYM	2.16	1.2	3.5	0.72	2.32	1.8	3.4	0.59	2.26	1.5	3.4	0.58
LYM (%)	30.63	14.5	42.3	8.9	31.1	19.7	43.8	6.65	30.68	14.9	38.8	6.60
MID	0.462	0.2	0.9	0.20	0.43	0.3	0.6	0.08	0.44	0.3	0.6	0.09
MID (%)	6.82	4.58	10	1.41	6.66	5.1	9.4	1.20	6.83	5.6	9.2	1.08
GRA	4.92	1.8	8.3	2.01	4.94	2.9	7.2	1.49	4.93	3.1	8.1	1.35
GRA (%)	61.52	50.6	82.7	10.04	62.23	49.6	75.2	7.25	62.48	54.4	79.5	6.99
RBC	4.56	4.13	5.36	0.419	4.61	4.25	5.42	0.39	4.66	4.28	5.38	0.38
HGB (g/dl)	12.7	10.6	15.2	1.49	12.73	10.4	15	1.56	12.84	9.8	15.7	1.87
HCT	38.6	33.1	43.9	3.9	38.5	31.3	45.1	4.29	38.5	29.2	46.9	5.07
MCV (fl)	85	65.4	94.2	8.20	83.71	60.6	93	9.41	83.51	60.5	92.7	9.39
MCH (pg)	28.11	20.9	31.8	3.09	27.68	20.1	31.5	3.31	27.95	20.4	31.7	3.46
MCHC (g/dl)	32.92	31.2	34.7	1.03	33.03	31.2	34	0.95	33.44	31	36	1.44
RDWa (fl)	44.33	12.9	56.8	17.2	52.7	37.5	60.4	6.48	52.38	38	60.7	6.44
RDWa (%)	13.01	11.6	16.5	1.52	13.07	12.1	14.4	0.88	13.1	11.8	14.4	1.01
PLT	234.9	136	341	66.9	239.6	150	319	59.5	234.8	149	320	49.6
MPV (fl)	9.26	8	10.2	0.72	9.12	7.7	10.1	0.87	9.14	8	10	0.63
PDWa (fl)	21.31	10.9	60.7	18.1	12.31	10.3	13.5	1.22	12.44	10.8	13.3	0.84
PDWa (%)	0.21	0.13	0.32	0.06	0.215	0.15	0.31	0.05	0.22	0.13	0.33	0.06
PCT	22.91	14.7	30.7	5.52	22.03	12.4	28.4	6.32	22.8	14.2	28.7	4.59
P-LCR	7.57	3.4	11.5	2.47	7.7	5.2	10.7	1.79	21.33	13	38	7.8
B. urea (mg/dl)	20.33	13	32	6.09	22.4	13	34	6.87	0.63	0.5	0.8	0.1
S. creatinine (mg/dl)	0.7	0.5	0.8	0.09	0.64	0.5	0.8	0.11	58.66	45	74	9.0
ALK. Phosphatase (IU/L)	56.33	43	74	11.74	49.11	39	59	7.97	19.13	14	23	3.02
GOT (AST) (IU/L)	18.22	14	22	2.31	21	13	33	6.21	17.86	9.8	44	10.1
GPT (ALT) (IU/L)	13.77	10	18	2.64	17.55	10	26	5.65	23.77	13	29	4.91
Gamma GT (GGT) (IU/L)	13.11	8	18	3.70	22.11	11	35	7.18	4.01	3.7	4.9	0.36
S. albumin (g/dL)	4.47	3.7	5.2	0.59	3.97	3.2	4.5	0.42	9.02	6.7	11.5	1.70
S. total protein (mg/dl)	7.077	6.4	8.4	0.78	9.44	6.8	10.4	0.80	9.64	9.1	10.17	0.35
Total calcium (mg/dl)	9.3	8.6	10.1	0.58	9.59	9.06	10.1	0.33	4.19	3.83	4.92	0.35
Potassium (mmol/l)	4.14	3.57	5.1	0.46	3.98	3.5	4.6	0.35	140.7	126	152.1	5.63
Sodium (mmol/l)	139.9	138	149.1	3.42	140.3	137.7	143.9	2.21	111.8	105.9	123.2	5.01
Chloride (mmol/l)	111.63	108	122.5	4.25	112.18	108	117.4	3.28	2.37	0.34	7.94	2.29
C-reactive protein (Mg/l)	2.08	0.13	6.99	2.57	2.43	0.04	10.76	3.18	7.63	5.1	10.1	1.47

**Table 2:** Comparison of blood parameters before and after 24 hours and 7 days following PRP injection.

	Blood markers	Normal level	Time	No.	Mean	SD	*F*-ratio	*p* value
1	WBC	4.0–10.0 10^9^/L	Tb0	18	7.578	2.47	0.01746	0.98
			Tb1		7.7	1.79		
			Tb7		7.633	1.47		
2	LYM	1.0–3.5 10^9^/L	Tb0	18	2.161	0.73	0.297	0.743
			Tb1		2.32	0.59		
			Tb7		2.267	1.5		
3	LYM%	2.0–45.0%	Tb0	18	30.7	8.9	0.0209	0.979
			Tb1		31.1	6.65		
			Tb7		30.7	6.6		
4	MID	0.2–1.0 10^9^/L	Tb0	18	0.47	0.2	0.199	0.819
			Tb1		0.43	0.08		
			Tb7		0.44	0.1		
5	MID%	2.0–10.0%	Tb0	18	6.84	1.42	0.1019	0.903
			Tb1		4.96	1.21		
			Tb7		61.5	1.09		
6	GRA	2.5–8.0 10^9^/L	Tb0	18	4.96	2.02	0.0007	0.999
			Tb1		4.94	1.5		
			Tb7		4.93	1.36		
7	GRA%	40–80%	Tb0	18	61.5	10.05	0.066	0.936
			Tb1		62.2	7.25		
			Tb7		62.5	6.99		
8	RBC	4.5–5.50 10^12^/L	Tb0	18	4.56	0.42	0.282	0.75
			Tb1		4.62	0.4		
			Tb7		4.66	0.38		
9	HGB	13.0–17.0 g/dl	Tb0	18	12.7	1.5	0.03	0.963
			Tb1		12.7	1.57		
			Tb7		12.8	1.87		
10	HCT	40–50%	Tb0	18	38.6	3.9	0.0037	0.996
			Tb1		38.5	4.29		
			Tb7		38.5	5.07		
11	MCV	80–100 fl	Tb0	18	85	8.2	0.144	0.865
			Tb1		83.7	9.4		
			Tb7		83.5	9.3		
12	MCH	27–32 pg	Tb0	18	28	3.09	0.077	0.925
			Tb1		27.7	3.32		
			Tb7		28	3.46		
13	MCHC	31–35 g/dl	Tb0	18	32.9	1.03	0.99	0.378
			Tb1		33	0.095		
			Tb7		33.4	1.44		
14	RDWa	30–150 fl	Tb0	18	44.4	17.3	3.17	0.50
			Tb1		52.7	6.48		
			Tb7		52.4	6.44		
15	RDWa%	11–16%	Tb0	18	13	1.53	0.027	0.972
			Tb1		13.1	0.89		
			Tb7		13.1	1.01		
16	PLT	100–400 10^9^/L	Tb0	18	235	66.9	0.038	0.962
			Tb1		240	59.6		
			Tb7		234	49.6		
17	MPV	7.0–11.0 fl	Tb0	18	9.24	0.73	0.178	0.837
			Tb1		9.12	0.87		
			Tb7		9.14	0.63		
18	PDWa	0.1–99.9 fl	Tb0	18	21.3	18.1	4.33	0.018^*∗*^
			Tb1		12.3	1.22		
			Tb7		12.4	0.84		
19	PCT	0.01–9.99%	Tb0	18	0.21	0.07	0.17	0.84
			Tb1		0.22	0.05		
			Tb7		0.23	0.06		
20	P-LCR	0.1–99.9%	Tb0	18	22.7	5.53	0.134	0.874
			Tb1		22	6.32		
			Tb7		22.8	4.59		
21	B. urea	16–48 mg/dl	Tb0	18	20.3	6.1	0.41	0.66
			Tb1		22.4	6.88		
			Tb7		21.3	7.81		
22	S. creatinine	0.6–1.3 mg/dl	Tb0	18	0.7	0.1	1.81	0.172
			Tb1		0.64	0.12		
			Tb7		0.63	0.12		
23	ALK. phosphate	20–140 IU/L	Tb0	18	56.3	11.7	4.74	0.013^*∗*^
			Tb1		49.1	7.98		
			Tb7		58.7	9.01		
24	GOT (AST)	<32 IU/L	Tb0	18	18.2	2.32	2.03	0.14
			Tb1		21	6.21		
			Tb7		19.1	3.02		
25	GPT (ALT)	<30 IU/L	Tb0	18	13.8	2.65	1.96	0.15
			Tb1		17.6	5.66		
			Tb7		17.9	10.2		
26	Gamma GT (GGT)	0–30 IU/L	Tb0	18	13.1	3.71	19.85	0.00001^*∗*^
			Tb1		22.1	7.19		
			Tb7		23.8	4.92		
27	S. albumin	3.5–5.2 g/dl	Tb0	18	4.48	0.6	6.33	0.0035^*∗*^
			Tb1		3.98	0.42		
			Tb7		4.01	0.36		
28	S. total protein	6.4–8.3 g/dl	Tb0	18	7.08	0.78	20.66	0.00001^*∗*^
			Tb1		9.44	0.8		
			Tb7		9.02	1.7		
29	Total calcium	8.6–10.2 mg/dl	Tb0	18	9.3	0.58	3.21	0.049^*∗*^
			Tb1		9.59	0.34		
			Tb7		9.65	0.35		
30	Potassium (K)	3.5–5.1 mmol/L	Tb0	18	4.14	0.46	1.47	0.239
			Tb1		3.98	0.35		
			Tb7		4.2	0.35		
31	Sodium (Na)	136–145 mmol/L	Tb0	18	140	3.43	0.179	0.836
			Tb1		140	2.22		
			Tb7		140	5.64		
32	Chloride (Cl)	95–115 mmol/L	Tb0	18	112	4.25	0.077	0.925
			Tb1		112	3.29		
			Tb7		112	5.01		
33	C-reactive protein (CRP)	<6.0 mg/L	Tb0		2.08	2.58	0.088	0.915
			Tb1		2.44	3.19		
			Tb7		2.38	2.29		

## Data Availability

The presented data are part of a Ph.D. thesis by Trefa Mohammad Ali Mahmood, College of Dentistry, University of Sulaimani, Kurdistan Region.

## Abbreviation

WBC: White blood cells
LYM: Lymphocyte
LYM%: Lymphocyte percentage
MID: Rare cells correlating to monocytes, eosinophils, basophils, blasts, and other precursor white cells that fall in a particular size range.
MID%: MID as percentage
GRA: Granulocytes (neutrophils, monocytes, basophils, and eosinophils)
GRA%: Granulocytes as percentage
RBC: Red blood cells
HGB: Hemoglobin
HCT: Hematocrit
MCV: Mean corpuscular volume
MCH: Mean corpuscular haemoglobin
MCHC: Mean corpuscular haemoglobin concentration
RDWa: Red blood cell distribution width average
RDWa%: Red cell distribution width as percentage
PLT: Platelets
MPV: Mean platelet volume
PDWa: Platelet distribution width
PDWa%: Platelet distribution width as percentage
PCT: Procalcitonin
P-LCR: Platelet larger cell ratio
B. urea: Blood urea
S. creatinine: Serum creatinine
ALK Phosphate: Alkaline phosphatase
GOT (AST): Aspartate aminotransferase
GPT (ALT): Aspartate aminotransferase
Gamma GT (GGT): Gamma-glutamyl transferase
S. albumin: Serum albumin
S. total protein: Serum total protein
CRP: C-reactive protein

## Additional Points

This is the first and only adequately powered study of the systemic effects of PRP. No previous study has been conducted revealing the effect of submucosal injection of PRP on the systemic blood markers which may interfere with some systemic diseases or cause other serious complications.
